# A first-in-class HBO1 inhibitor WM-3835 inhibits castration-resistant prostate cancer cell growth in vitro and in vivo

**DOI:** 10.1038/s41419-023-05606-5

**Published:** 2023-01-28

**Authors:** Yuan-yuan Mi, Yu Ji, Lifeng Zhang, Chuan-yu Sun, Bing-bing Wei, Dong-jie Yang, Hong-yuan Wan, Xiao-wei Qi, Sheng Wu, Li-jie Zhu

**Affiliations:** 1 Department of Urology, Affiliated Hospital of Jiangnan University, Wuxi, China; 2 Department of Pathology, Affiliated Hospital of Jiangnan University, Wuxi, China; 3 Department of Urology, Affiliated Changzhou No. 2 People’s Hospital of Nanjing Medical University, Changzhou, China; 4 Department of Urology, Huashan Hospital, Fudan University, Shanghai, China; 5 Department of Urology, Wuxi People’s Hospital of Nanjing Medical University, Wuxi, China

**Keywords:** Targeted therapies, Prostate cancer

## Abstract

The prognosis and overall survival of castration-resistant prostate cancer (CRPC) patients are poor. The search for novel and efficient anti-CRPC agents is therefore extremely important. WM-3835 is a cell-permeable, potent and first-in-class HBO1 (KAT7 or MYST2) inhibitor. Here in primary human prostate cancer cells-derived from CRPC patients, WM-3835 potently inhibited cell viability, proliferation, cell cycle progression and in vitro cell migration. The HBO1 inhibitor provoked apoptosis in the prostate cancer cells. It failed to induce significant cytotoxicity and apoptosis in primary human prostate epithelial cells. shRNA-induced silencing of HBO1 resulted in robust anti-prostate cancer cell activity as well, and adding WM-3835 failed to induce further cytotoxicity in the primary prostate cancer cells. Conversely, ectopic overexpression of HBO1 further augmented primary prostate cancer cell proliferation and migration. WM-3835 inhibited H3-H4 acetylation and downregulated several pro-cancerous genes (*CCR2*, *MYLK*, *VEGFR2*, and *OCIAD2*) in primary CRPC cells. Importantly, *HBO1* mRNA and protein levels are significantly elevated in CRPC tissues and cells. In vivo, daily intraperitoneal injection of WM-3835 potently inhibited pPC-1 xenograft growth in nude mice, and no apparent toxicities detected. Moreover, intratumoral injection of HBO1 shRNA adeno-associated virus (AAV) suppressed the growth of primary prostate cancer xenografts in nude mice. H3-H4 histone acetylation and HBO1-dependent genes (*CCR2*, *MYLK*, *VEGFR2*, and *OCIAD2*) were remarkably decreased in WM-3835-treated or HBO1-silenced xenograft tissues. Together, targeting HBO1 by WM-3835 robustly inhibits CRPC cell growth.

## Introduction

Prostate cancer is one of the most common malignant tumors among men worldwide [1, 2]. In developed countries, the incidence of prostate cancer ranks first among male population. The mortality rate of prostate cancer in the United States ranks second among male malignancies [1, 2]. In addition, in developing countries such as China, the incidence of prostate cancer is increasing recently, and a significant number of patients are already in the middle/late stages after diagnose [3, 4].

Current therapies, including prostatectomy, androgen deprivation therapy (ADT), and radiotherapy, can control and improve the condition of patients with advanced prostate cancer, but only within a certain period of time [3, 4]. After a median remission period of 18–24 months, prostate cancer patients can progress to metastatic castration-resistant prostate cancer (CRPC). These patients often have poor prognosis [3, 4] and the median survival is only about 12 months [3, 4]. Docetaxel is the first-line chemotherapeutic drug for the treatment of CRPC, but a large proportion of patients can eventually develop resistance [3, 4]. The U.S. Food and Drug Administration (FBA) has approved the new endocrine drugs, enzalutamide, and abiraterone, for the treatment of CRPC before or after failure of docetaxel chemotherapy [5–7]. However, the prognosis for CRPC is still far from satisfactory [5–7].

Multiple molecular signaling targets, including androgen receptor (AR) and others, play an important role in the development and progression of CRPC and other advanced prostate cancers [8]. Epigenetic dysregulation is critically involved in the pathogenesis and progression of CRPC [9]. Histone acetylation, controlled by multiple histone deacetylases (HDAC) and histone acetyltransferases (HAT), is known as an important epigenetic mechanism regulating gene expression [10–12]. HBO1 (KAT7 or MYST2) can associate with acetyl-CoA and catalyze acetylation of H4 and H3 histones at various lysine residues [13, 14]. HBO1 also binds to different subunits and cofactors, important in gene transcription and regulating multiple physiological functions and behaviors [14–19]. Recent studies have established an important role of HBO1 in the development and progression of multiple malignancies [14, 20–24]. Inactivation, silencing, or depletion of HBO1 could result in robust anti-cancer activity [14, 20–24].

WM-3835 is a first-in-class HBO1 inhibitor that hinders Histone H3/H4 acetylation and downregulates multiple genes that are closely associated with cancer progression [20, 25]. The results of this study will show that HBO1 inactivation by WM-3835 robustly inhibits primary CRPC cell growth in vitro and in vivo.

## Materials and methods

### Reagents

WM-3835, all the antibodies, viral constructs, and mRNA primers were provided by Dr. Cao at Soochow University [25]. The caspase inhibitors zDEVD-fmk and zVAD-fmk, as well as the cell counting kit-8 (CCK-8) kit, docetaxel, and lactate dehydrogenase (LDH) assay kit were purchased from Sigma (St. Louis, MO). All fluorescence dyes were purchased from Thermo-Fisher Invitrogen (Shanghai, China).

### Primary human cells

The fresh tumor tissues and matched adjacent epithelial tissues of different CRPC patients were first cut into small pieces and digested by using collagenase I plus dispase II combo. The resulting primary cells were maintained under the complete medium and were filtered. Vascular and endothelial cells, fibroblasts, and immune cells, floating in the supernatant, were abandoned. The primary cancer cells or prostate epithelial cells were cultivated in the descried medium [26]. Here, the primary human prostate cancer cells (“pPC-1”, “pPC-2”, “pPC-3”, and “pPC-4”, derived from four written-informed consent CRPC patients) and the primary human prostate epithelial cells (“pEpi1” and “pEpi2”, derived from two patients) were obtained. The protocols of using human cells were approved by the Ethics Board of Affiliated Hospital of Jiangnan University.

### Human tissues

Ten CRPC patients (all male, 59 to 82-year old), administrated at Affiliated Hospital of Jiangnan University, were enrolled. The written-informed consent was obtained from each patient. Fresh CRPC tumor tissues (at primary site) and matched adjacent normal prostate tissues were obtained and separately carefully. Tissues were kept in liquid nitrogen before further analyses. The protocols were approved by Affiliated Hospital of Jiangnan University’s Ethics Committee and were in according to Declaration of Helsinki.

### HBO1 silencing

The HBO1-shRNA-expresssing lentivirus or scramble control shRNA lentivirus (“shC”) were provided by Dr. Cao at Soochow University [25] and were added to the primary cultured prostate cancer cells or epithelial cells. After 72 h, cells were maintained in fresh complete medium, and puromycin was added. After 48 h, the stable cells were established, and *HBO1* silencing was verified. For the in vivo studies, adeno-associated virus (AAV)-packed HBO1 shRNA (“aav-shHBO1-Sq1”) or scramble control shRNA, again from Dr. Cao [25], were injected intratumorally to xenograft-bearing mice.

### HBO1 overexpression

The HBO1-cDNA-expresssing lentiviral construct or vector control (“Vec”) were provided by Dr. Cao at Soochow University [25] and were transduced to the primary cultured prostate cancer cells or epithelial cells. After 72 h, cells were maintained in fresh complete medium, and puromycin was added. After 4-5 passages, the stable cells were established and overexpression of HBO1 was verified.

### Other assays

Western blotting, real-time quantitative reverse transcription PCR (qRT-PCR), nuclear EdU (5-Ethynyl-2′- deoxyuridine)/DAPI (4′,6-diamidino2-phenylindole) staining (testing cell proliferation), nuclear TUNEL (TdT-mediated dUTP Nick-End Labeling)/DAPI staining, propidium iodide (PI)-flow cytometry (testing cell cycle progression), “Transwell” assays (testing in vitro cell migration) and Caspase-3/−9 activity assays were described in other studies [20, 27–30]. Figure S1 listed the uncropped blotting images.

### Animal xenograft studies

The nude mice, all male, at 17.8–18.3 g, were provided by Shanghai Laboratory Animal Center (SLAC, Shanghai, China). The primary pPC-1 cells, at 7.5 × 10^6^ cells per tumor, were subcutaneously (*s.c*.) injected to the flanks of the nude mice. The subcutaneous pPC-1 xenograft tumors were thereafter formed, and their volumes were close to 100 mm^3^ at 20 days after cell injection (“Day-1”). The xenograft-bearing nude mice were then intraperitoneally injected with WM-3835 (at 5 mg/kg body weight, daily for 14 days) or the vehicle control [25]. Alternatively, adeno-associated virus (AAV)-packed HBO1 shRNA (“aav-shHBO1-Sq1”) or AAV-packed scramble control shRNA were intratumorally injected to the xenografts. AAV injection was carried out daily for 10 days. Tumor volumes, mice body weights, and estimated daily tumor growth (in mm^3^ per day) was calculated as described [31]. All animal studies were approved by the IACUC and Institute Animal Ethics Review Board of Affiliated Hospital of Jiangnan University.

### Statistical analysis

In vitro experiments were repeated five times. Data were presented as mean ± standard deviation (SD). Statistical analyses were performed under the SPSS 23.0 software (SPSS Co., Chicago, IL). Unpaired student’s T-test was utilized when comparing two groups. One-way ANOVA plus the Scheffe’ and Tukey Test was carried out for comparison three and more groups. *P* values of <0.05 were considered as statistically significant. IC-50 (the concentration resulting in 50% CCK-8 viability reduction) was calculated by nonlinear regression analysis using GraphPad Prism 5.01.

## Results

### WM-3835 inhibits primary CRPC cell viability, cell cycle progression, proliferation and migration in vitro

**Fig. 1:**
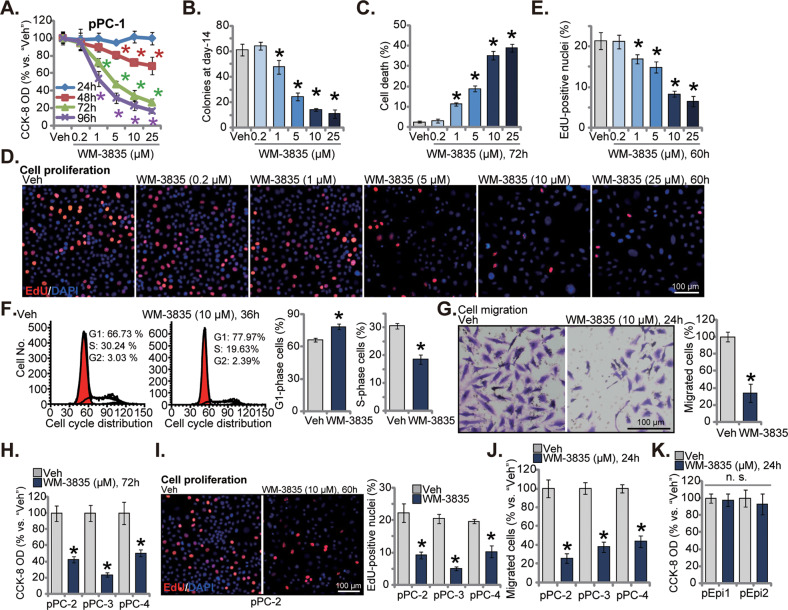
WM-3835 inhibits primary CRPC cell viability, cell cycle progression, proliferation and migration in vitro. The primary human prostate cancer cells derived from a CRPC patient, “pPC-1”, were treated with WM-3835 (at designated concentrations) or vehicle control (“Veh”) for designated hours, cell viability (CCK-8 assay, **A**), colony formation (**B**), cell death (by testing medium LDH releasing, **C**) and proliferation (by testing EdU incorporation, **D** and **E**), as well as cell cycle distribution (**F**) and in vitro cell migration (**G**) were tested. The primary human prostate cancer cells derived from three other CRPC patients, “pPC-2/pPC-3/pPC-4” (**H**–**J**), or the primary human prostate epithelial cells (“pEpi1” and “pEpi2”, derived from two patients) (**K**) were treated WM-3835 (10 μM) or vehicle control (“Veh”) for designated hours, cell viability (**H**, **K**), proliferation (**I**), and migration (**J**) were examined similarly. Data were expressed as the mean ± standard deviation (SD, n = 5). *P < 0.05 versus “Veh” group. “n. s.” stands for non-statistical difference (*P* > 0.05). Scale bar = 100 μm.

The primary human prostate cancer cells derived from a CRPC patient, pPC-1, were cultivated in complete medium and treated with the HBO1 inhibitor WM-3835. CCK-8 assay results, Fig. 1A, revealed that the HBO1 inhibitor robustly inhibited viability of pPC-1 cells. WM-3835 showed a dose-dependent response in decreasing the viability of pPC-1 cells (Fig. 1A). CCK-8 viability OD (optic density) reduction was significant after 1–25 μM of WM-3835 treatment (Fig. 1A). It was however ineffective at 0.2 μM (Fig. 1A). The viability reduction by WM-3835 was time-dependent as well. It took at least 48 h for the HBO1 inhibitor to impose significant anti-survival effect (Fig. 1A). Further, the HBO1 inhibitor dose-dependently decreased the number of viable pPC-1 cell colonies (Fig. 1B). Moreover, WM-3835, at 1–25 μM, provoked pPC-1 cell death and induced significant medium LDH releasing (Fig. 1C). It also robustly suppressed pPC-1 cell proliferation, as the EdU-positive nuclei ratio was significantly decreased following 1–25 μM of WM-3835 treatment (Fig. 1D, E). Therefore WM-3835 dose-dependently inhibited pPC-1 cell survival and proliferation (Fig. 1A–E). The titration experimental results revealed that WM-3835 at 10 μM caused robust anti-pPC-1 cell activity (Fig. 1A–E), this concentration was close to IC-50 (6.24 ± 0.65 μM) and was selected for the following studies (Fig. 1A–E).

Next the PI-FACS assays were carried out to study the potential effect of WM-3835 on cell cycle progression. WM-3835 (10 μM, 36 h) treatment in pPC-1 cells increased G1 phase percentage while decreasing S-phase percentage (Fig. 1F). Therefore, the HBO1 inhibitor caused G1-S arrest in pPC-1 cells (Fig. 1F). Moreover, WM-3835 (10 μM) inhibited in vitro migration (Fig. 1G) of pPC-1 cells, based on the results from “Transwell” assay. The potential effect of WM-3835 in other cancer cells was studied as well. The primary human prostate cancer cells that were derived from three other CRPC patient, “pPC-2/pPC-3/pPC-4”, were treated with WM-3835 (10 μM). As shown, in these primary cancer cells, the HBO1 inhibitor robustly decreased CCK-8 viability (Fig. 1H), EdU incorporation (Fig. 1I), and slowed cell migration (Fig. 1J). Importantly, treatment with WM-3835 (5 μM) significantly potentiated docetaxel (15 nM)-induced viability reduction and cell death in pPC-1 and pPC-2 cells (Figure S2A, B). On the contrast, in the primary human prostate epithelial cells (“pEpi1” and “pEpi2”), treatment with WM-3835 (10 μM) failed to significantly inhibit CCK-8 viability (Fig. 1K).

### WM-3835 provokes apoptosis in primary CRPC cells

**Fig. 2:**
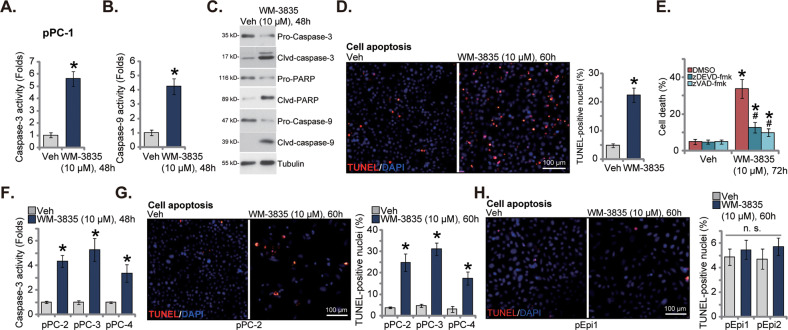
WM-3835 provokes apoptosis in primary CRPC cells. The primary human prostate cancer cells derived from a CRPC patient, “pPC-1”, were treated with WM-3835 (10 μM) or vehicle control (“Veh”) for designated hours, caspase-PARP activation was measured (**A**–**C**); Cell apoptosis was tested by nuclear TUNEL staining assay (**D**). pPC-1 cells were pretreated with zDEVD-fmk (45 μM), zVAD-fmk (45 μM), or DMSO (0.1%) for 1 h, followed by WM-3835 (10 μM) treatment for another 72 h, cell death was tested by LDH releasing assay (**E**). The primary human prostate cancer cells derived from three other CRPC patients, “pPC-2/pPC-3/pPC-4” (**F**, **G**), or the primary human prostate epithelial cells (“pEpi1” and “pEpi2”, derived from two patients) (**H**) were treated WM-3835 (10 μM) or vehicle control (“Veh”) for designated hours, caspase-3 activity (**F**), and apoptosis (**G**, **H**) were tested using the similar methods. Data were expressed as the mean ± standard deviation (SD, n = 5). *P < 0.05 versus “Veh” group. ^#^*P* < 0.05 versus “DMSO” treatment (**E**). “n. s.” stands for non-statistical difference (*P* > 0.05). Scale bar = 100 μm.

In prostate cancer cells, growth inhibition and cell cycle arrest can provoke apoptosis [32–34]. Recent studies have discovered that HBO1 silencing or inhibition could cause apoptosis activation in different human cancer cells [21, 25]. We therefore examined the potential effect of WM-3835 on cell apoptosis. In pPC-1 primary cells, WM-3835 (10 μM) robustly increased the activities of caspase-3 and caspase-9 (Fig. 2A and B). Cleavages of caspase-3, caspase-9 and poly (ADP-ribose) polymerase (PARP) were increased in WM-3835-stimulated pPC-1 cells (Fig. 2C), whereas pro-caspase-3, pro-caspase-9 and pro-PARP levels were decreased (Fig. 2C). Following treatment of WM-3835, the TUNEL-positive nuclei ratio was significantly increased in pPC-1 cells (Fig. 2D), indicating apoptosis activation. Importantly, caspase inhibitors, including zDEVD-fmk (a caspase-3 specific inhibitor) and zVAD-fmk (a pan caspase inhibitor) largely inhibited WM-3835 (10 μM)-induced pPC-1 cell death (Fig. 2E). Thus, caspase-apoptosis activation should be the primary mechanism of WM-3835-induced cytotoxicity in CRPC cells.

In the primary pPC-2/pPC-3/pPC-4 CRPC cells, WM-3835 provoked significant apoptosis. The HBO1 inhibitor increased the caspase-3 activity (Fig. 2F) and TUNEL-positive nuclei percentage (Fig. 2G) in the primary cancer cells. The HBO1 inhibitor however failed to induce apoptosis in the epithelial cells and the TUNEL percentage was unchanged following treatment of WM-3835 in pEpi1 and pEpi2 epithelial cells (Fig. 2H). Thus, WM-3835 specifically provoked apoptosis in primary CRPC cells.

### HBO1 silencing inhibits primary CRPC cell viability, proliferation and migration in vitro

**Fig. 3:**
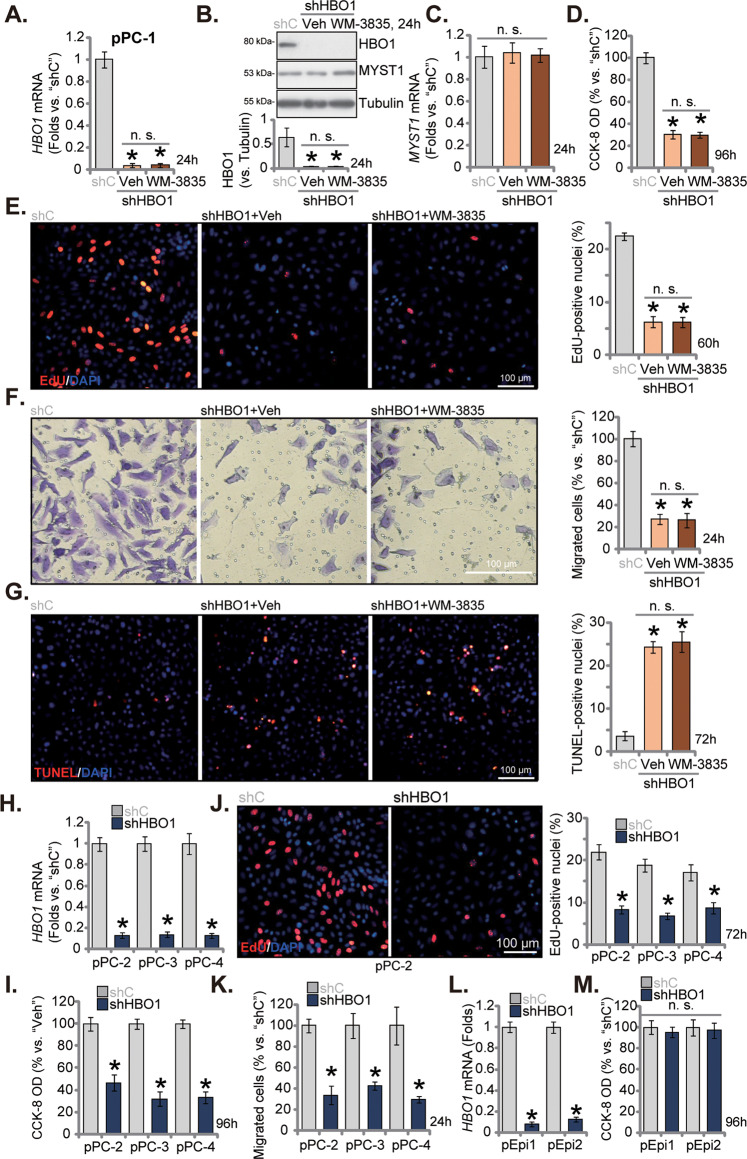
HBO1 silencing inhibits primary CRPC cell viability, proliferation and migration in vitro. The primary pPC-1 cells were infected with the HBO1-shRNA-expressing lentivirus (“shHBO1”) or the scramble control shRNA-expressing lentivirus (“shC”), and stable cells were formed after selection and verification. The shHBO1 pPC-1 cells were further treated with WM-3835 (10 μM) or vehicle control (“Veh”) for designated hours, expression of listed mRNAs and proteins was measured (**A**–**C**); Cell viability, proliferation, in vitro migration and apoptosis were tested by CCK-8 (**D**), the nuclear EdU incorporation staining (**E**), “Transwell” (**F**) and nuclear TUNEL staining (**G**) assays, respectively. The primary human prostate cancer cells derived from three other CRPC patients, pPC-2/pPC-3/pPC-4 (**H**–**K**), or the primary human prostate epithelial cells (“pEpi1” and “pEpi2”, derived from two patients) (**L**, **M**), were stably transduced with shHBO1 or shC, *HBO1* mRNA expression was measured (**H**, **L**); After culturing for indicated time periods, cell viability (**I**, **M**), cell proliferation (**J**) and in vitro cell migration (**K**) were measured similarly. Data were expressed as the mean ± standard deviation (SD, n = 5). *P < 0.05 versus “shC” group. “n. s.” stands for non-statistical difference (*P* > 0.05). Scale bar = 100 μm.

We further hypothesized that HBO1 depletion, using genetic means, should mimic WM-3835-induced anti-CRPC cell actions. Therefore, the HBO1-shRNA-expressing lentivirus (from Dr. Cao [25]) was added to pPC-1 primary cells. The stable cells, namely “shHBO1” cells, were thereafter formed following puromycin selection. As compared to the control cells with the scramble control shRNA (“shC”), the applied HBO1 shRNA resulted in remarkable *HBO1* mRNA (Fig. 3A) and protein (Fig. 3B) silencing. *MYST1* mRNA (Fig. 3C) and protein (Fig. 3B) expression was unchanged. shRNA-induced silencing of HBO1 decreased CCK-8 viability (Fig. 3D) and inhibited proliferation (EdU incorporation, Fig. 3E). Moreover, in vitro cell migration (Fig. 3F) of the shHBO1 pPC-1 cells was significantly slowed. Importantly, in HBO1-silenced pPC-1 cells, adding WM-3835 (10 μM) failed to further inhibit cell viability (Fig. 3D), proliferation (Fig. 3E) and in vitro cell migration (Fig. 3F). Moreover, HBO1 silencing provoked apoptosis and increased TUNEL-nuclei percentage in pPC-1 cells (Fig. 3G), which was not significantly affected by WM-3835 (10 μM) treatment (Fig. 3G). These results supported that WM-3835-induced anti-CRPC cell activity was due to HBO1 inhibition.

In the primary pPC-2/pPC-3/pPC-4 cells, stably transducing the lentiviral HBO1 shRNA resulted in robust HBO1 *mRNA* downregulation as well (Fig. 3H). HBO1 silencing also inhibited cell viability (Fig. 3I), proliferation (EdU incorporation, Fig. 3J) and slowed in vitro cell migration (Fig. 3K) in these primary CRPC cells. On the other hand, in pEpi1 and pEpi2 primary epithelial cells, shRNA-induced silencing of HBO1 (Fig. 3L) failed to significantly decrease CCK-8 viability (Fig. 3M).

### Ectopic overexpression of HBO1 exerts pro-cancerous activity in primary CRPC cells

**Fig. 4:**
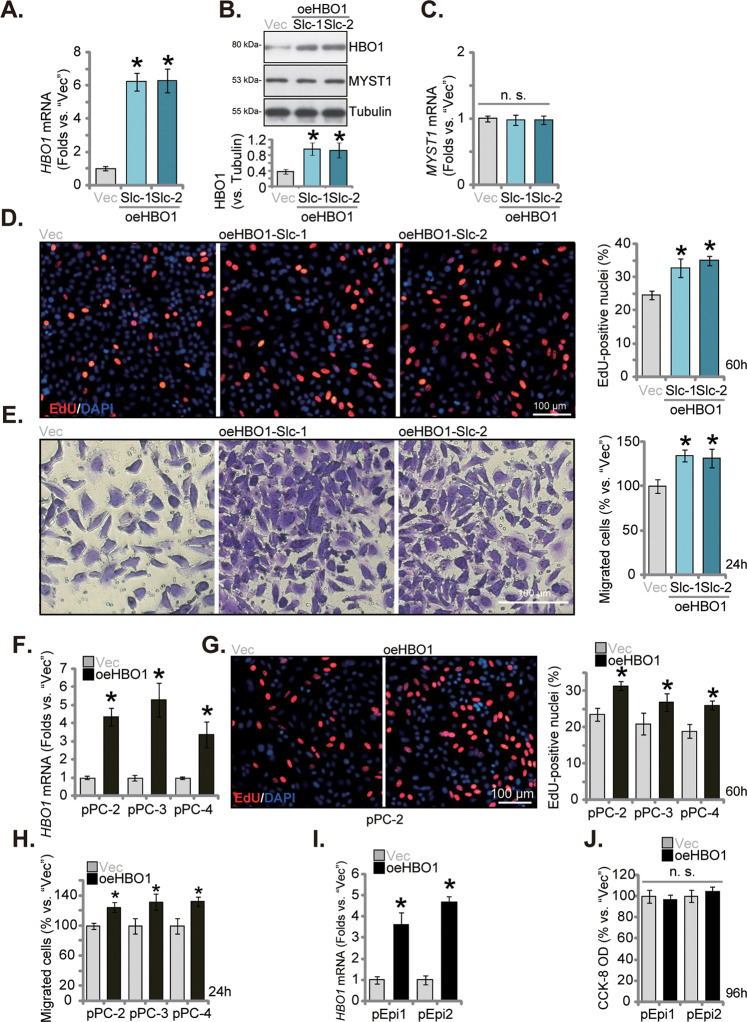
Ectopic overexpression of HBO1 exerts pro-cancerous activity in primary CRPC cells. The primary pPC-1 cells were transduced with a lentiviral HBO1-expressing construct, and two stable selections, oeHBO1-Slc-1 and oeHBO1-Slc-2, were formed after selection and overexpression verification. Control pPC-1 cells were stably transduced with the empty vector (“Vec”); Expression of *HBO1* mRNA and listed proteins was tested (**A**–**C**). Cells were cultivated for the designated hours, and cell proliferation and in vitro cell migration were tested by nuclear EdU incorporation (**D**) and “Transwell” (**E**) assays, respectively. The primary human prostate cancer cells derived from three other CRPC patients, pPC-2/pPC-3/pPC-4 (**F**–**H**), or the primary human prostate epithelial cells (“pEpi1” and “pEpi2”, derived from two patients) (**I**, **J**), were stably transduced with the lentiviral HBO1-expressing construct (“oeHBO1”) or the empty vector (“Vec”), *HBO1* mRNA expression was measured (**F**, **I**). After culturing for indicated time periods, cell proliferation (**G**), in vitro migration (**H**) and cell viability (**J**) were measured similarly. Data were expressed as the mean ± standard deviation (SD, *n* = 5). *P < 0.05 versus “Vec” group. “n. s.” stands for non-statistical difference (*P* > 0.05). Scale bar = 100 μm.

We have shown that WM-3835 treatment or HBO1 silencing resulted in significant anti-CRPC cell activity. Ectopic overexpression of HBO1 should then exert opposite functions. Therefore, a lentiviral HBO1-expressing construct (from Dr. Cao [25]) was transduced to pPC-1 primary cells. Following selection by puromycin and HBO1 expression verification, two selections of HBO1-overexpressed pPC-1 cells were formed, namely “oeHBO1-Slc-1” and “oeHBO1-Slc-2”. As compared the vector control cells (“Vec”), *HBO1* mRNA (Fig. 4A) and protein (Fig. 4B) levels were remarkably increased in oeHBO1-Slc-1 and oeHBO1-Slc-2 cells. *MYST1* mRNA (Fig. 4C) and protein (Fig. 4B) expression was unchanged. Ectopic overexpression of HBO1 increased cell proliferation (Fig. 4D) in pPC-1 cells. The in vitro cell migration (Fig. 4E) was accelerated following HBO1 overexpression in the pPC-1 cells.

In the primary pPC-2/pPC-3/pPC-4 cells, stably transducing the lentiviral HBO1-expressing construct similarly resulted in *HBO1* mRNA upregulation (“oeHBO1”, Fig. 4F). Functional studies revealed that oeHBO1 augmented cell proliferation (EdU incorporation, Fig. 4G) and speeded in vitro cell migration (Fig. 4H) in the primary CRPC cells. To the pEpi1 and pEpi2 primary epithelial cells, the lentiviral HBO1-expressing construct was stably transduced, causing robust *HBO1* mRNA upregulation (“oeHBO1”) (Fig. 4I); However, HBO1 overexpression failed to affect the viability of the primary epithelial cells (Fig. 4J). These results again supported the oncogenic function of HBO1.

### WM-3835 inhibits H3-H4 acetylation and expression of several pro-cancerous genes in primary CRPC cells

**Fig. 5:**
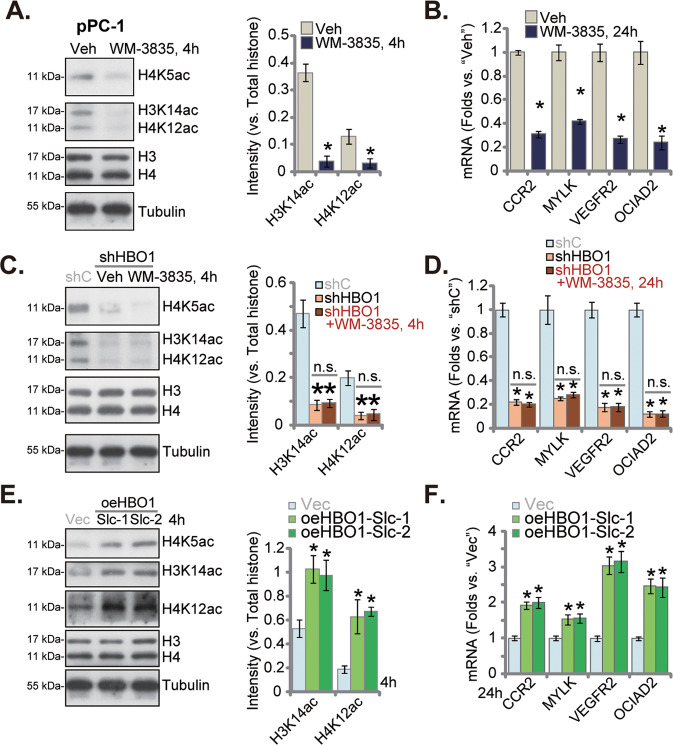
WM-3835 inhibits H3-H4 acetylation and expression of several pro-cancerous genes in primary CRPC cells. The primary pPC-1 cells were treated with WM-3835 (10 μM) or vehicle control (“Veh”) for designated hours, expression of listed proteins and mRNAs were tested (**A** and **B**). The stable primary pPC-1 cells with HBO1 shRNA (“shHBO1”) or the scramble control shRNA (“shC”) were further treated with or without WM-3835 (10 μM) for designated hours, expression of listed proteins and mRNAs were tested (**C** and **D**); The primary pPC-1 cells, with the lentiviral HBO1-expressing construct (oeHBO1-Slc-1 and oeHBO1-Slc-2, two stable selections) or the empty vector (“Vec”) were established, expression of listed proteins and mRNAs were tested (**E** and **F**); Data were expressed as the mean ± standard deviation (SD, n = 5). *P < 0.05 versus “Veh”/“shC”/“Vec” group. “n. s.” stands for non-statistical difference (*P* > 0.05).

HBO1 is vital for histone H3/H4 acetylation and expression of multiple oncogenic/pro-cancerous genes [20, 21, 24]. Here, in the pPC-1 primary cells, treatment with WM-3835 (10 μM, 4 h) robustly decreased H3K14 acetylation (H3K14ac) and H4 acetylation (H4K5ac and H4K12ac, at two different sites) (Fig. 5A). Total H3 and H4 protein levels were unchanged (Fig. 5A). mRNA expression of HBO1-dependent genes, including *C‑C chemokine receptor type 2* (*CCR2*), *myosin light chain kinase* (*MYLK*), *vascular endothelial growth factor receptor 2* (*VEGFR2*), *ovarian cancer immunoreactive antigen domain containing 2* (*OCIAD2*) [20, 21, 24], were robustly decreased in WM-3835-treated cells (Fig. 5B). Moreover, shRNA-caused silencing of HBO1 (see Fig. 3) similarly decreased H3K14ac, H4K5ac and H4K12ac (Fig. 5C), and downregulated *CCR2*, *MYLK*, *VEGFR2* and *OCIAD2* mRNA levels (Fig. 5D). Adding WM-3835 failed to further augment HBO1 silencing-induced actions (Fig. 5C, D). Contrarily, in HBO1-overexpressed pPC-1 primary cells, oeHBO1-Slc-1 and oeHBO1-Slc-2 (see Fig. 4), H3/H4 acetylation (Fig. 5E) and expression of *CCR2*, *MYLK*, *VEGFR2* and *OCIAD2* (Fig. 5F) were significantly increased. WM-3835 inhibited H3-H4 acetylation and several pro-cancerous genes expression in CRPC cells.

### HBO1 overexpression in CRPC cells and tissues

**Fig. 6:**
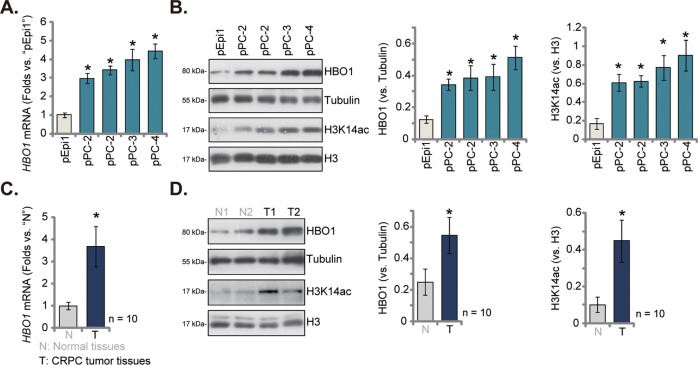
HBO1 overexpression in CRPC cells and tissues. Expression of *HBO1* mRNA (**A**, **C**) and listed proteins (**B**, **D**) in primary human prostate epithelial cells (“pEpi1”), the listed primary CRPC cells (“pPC-1/pPC-2/pPC-3/pPC-4”), the CRPC tumor tissues (“T”, derived from 10 different CRPC patients, *n* = 10) or tumor-surrounding normal prostate tissues (“N”) were shown. Data were expressed as the mean ± standard deviation (SD). *P < 0.05 versus “pEpi1” cells or “N” tissues.

We also examined the expression level of HBO1 in CRPC cells and tissues. As demonstrated, *HBO1* mRNA expression in primary prostate cancer cells that were derived from four different CRPC cells (“pPC-1/pPC-2/pPC-3/pPC-4”) was significantly higher than that in the primary human prostate epithelial cells (“pEpi1”) (Fig. 6A). HBO1 protein upregulation was also detected in the primary CRPC cells (Fig. 6B), where H3K14ac levels were increased (Fig. 6B). Further studies demonstrated that *HBO1* mRNA and protein levels were significantly upregulated in human CRPC tumor tissues that were derived from 10 different CRPC patients (Fig. 6C, D). Whereas their expression was relatively low in the tumor-surrounding normal prostate tissues (Fig. 6C, D). H3K14ac levels were elevated in CRPC tumor tissues as well (Fig. 6D). These results confirmed HBO1 overexpression in CRPC cells and tissues.

### WM-3835 injection hinders pPC-1 xenograft growth in nude mice

**Fig. 7:**
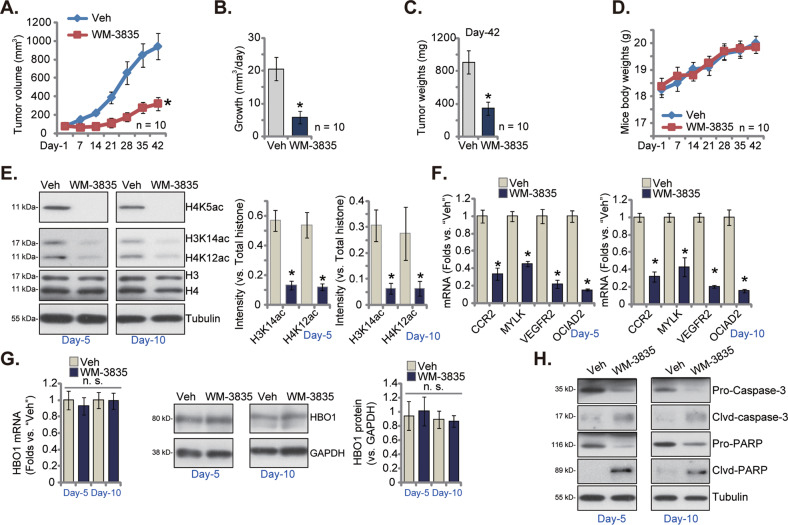
WM-3835 injection hinders pPC-1 xenograft growth in nude mice. The pPC-1 xenograft-bearing nude mice were intraperitoneally (*i.p*.) injected with WM-3835 (at 5 mg/kg body weight, daily for 14 days) or the vehicle control (“Veh”), tumor volumes (**A**) and mice body weights (**D**) were recorded every seven days (“Day-1” to “Day-42”), daily tumor growth (in mm^3^ per day, **B**) and tumor weights (at Day-42, **C**) were measured as well. Expression of listed genes and proteins in the listed pPC-1 xenograft tissue lysates was tested (**E**–**H**). Data were expressed as the mean ± standard deviation (SD). *P < 0.05 versus “Veh” group. “n. s.” stands for non-statistical difference (*P* > 0.05).

Next experiments were carried out to study the potential activity of WM-3835 in vivo. The primary pPC-1 cells were *s.c*. injected to the flanks of nude mice and pPC-1 xenografts were formed after 20 days (labeled as “Day-1”). The pPC-1 xenograft mice were thereafter randomly assigned into two groups and were *i.p*. injected with WM-3835 or the vehicle control (“Veh”). WM-3835 was administrated at 5 mg/kg body weight, daily for 14 consecutive days. The weekly tumor growth curve results, Fig. 7A, revealed that WM-3835 administration robustly hindered pPC-1 xenograft growth in nude mice, as the volumes of WM-3835-treated pPC-1 xenografts were remarkably lower than the control xenografts with vehicle administration (Fig. 7A). Figure 7B calculated the estimated daily tumor growth (in mm^3^ per day) and showed again that WM-3835 treatment potently inhibited pPC-1 xenograft growth. At Day-42 the pPC-1 xenografts were carefully resected from the mice and individually weighted. The WM-3835-treated pPC-1 xenografts were significantly lighter than those with vehicle treatment (Fig. 7C). We failed to observe any apparent toxicities in mice of both groups and the mice body weights were not significantly different between control and WM-3835 groups (Fig. 7D). These results showed that daily *i.p*. injection of WM-3835 in mice robustly hindered pPC-1 xenograft growth.

At treatment Day-5 and Day-10, 6 h after WM-3835/vehicle administration, one pPC-1 xenograft per group was resected, and a total of four xenografts were obtained. Each xenograft was cut into five small pieces, dissolved in tissue lysis buffer, and tested individually. As shown levels of H4K5ac, H4K12ac and H3K14ac were robustly decreased in WM-3835-administrated pPC-1 xenograft tissues (Fig. 7E). HBO1-dependent mRNAs, including *CCR2*, *MYLK*, *VEGFR2* and *OCIAD2*, were downregulated following WM-3835 administration (Fig. 7F). *HBO1* mRNA and protein expression was however unchanged following WM-3835 treatment (Fig. 7G). Cleaved-caspase-3 and cleaved-PARP were significantly increased WM-3835-treated pPC-1 xenograft tissues (Fig. 7H), where pro-caspase-3 and pro-PARP were decreased (Fig. 7H). Thus, WM-3835 administration provoked apoptosis activation in pPC-1 xenografts.

### HBO1 silencing hinders pPC-1 xenograft growth in nude mice

**Fig. 8:**
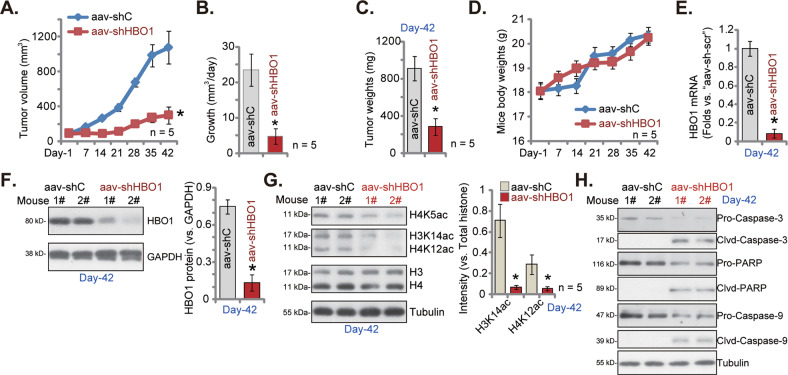
HBO1 silencing hinders pPC-1 xenograft growth in nude mice. The pPC-1 xenograft-bearing nude mice were intratumorally injected with AAV-packed HBO1 shRNA (“aav-shHBO1”) or AAV-packed scramble control shRNA (“aav-shC”). AAV was injected daily for 10 consecutive days. Tumor volumes (**A**) and mice body weights (**D**) were recorded every seven days (“Day-1” to “Day-42”), daily tumor growth (in mm^3^ per day, **B**) and tumor weights (at Day-42, **C**) were measured as well. Expression of listed genes and proteins in the listed pPC-1 xenograft lysates were tested (**E**, **H**). Data were expressed as the mean ± standard deviation (SD). *P < 0.05 versus “aav-shC” group.

To further support the important role of HBO1 on CRPC cell growth, the pPC-1 xenograft-bearing mice were treated with AAV-packed HBO1 shRNA (“aav-shHBO1”) or AAV-packed scramble control shRNA (“aav-shC”). AAV was intratumorally injected, daily for 10 days. Tumor volumes were again recorded. Results revealed that aav-shHBO1 injection potently inhibited pPC-1 xenograft growth in nude mice (Fig. 8A) and significantly hindered daily tumor growth (Fig. 8B). At Day-42, pPC-1 xenografts were again separated carefully and weighted together. As compared to aav-shC-treated group, aav-shHBO1-injected tumors were significantly lighter (Fig. 8C). The mice body weights were again not significantly different between aav-shHBO1 and aav-shC groups (Fig. 8D). Tumors were homogenized and dissolved in tissue lysis buffer and signaling proteins were tested. *HBO1* mRNA and protein expression was indeed silenced in aav-shHBO1-injected tumors (Fig. 8E, F), where levels of H4K5ac, H4K12ac, and H3K14ac were decreased (Fig. 8G). Cleaved-caspase-3 and cleaved-PARP levels were increased in pPC-1 xenografts with aav-shHBO1 injection (Fig. 8H), and pro-caspase-3 and pro-PARP decreased. These results showed that HBO1 silencing inhibited primary CRPC cell growth in vivo.

## Discussion

Endocrine therapy is the primary treatment for advanced prostate cancer [3, 4]. Yet most patients will gradually develop CRPC after 14-20 months of treatment, with the median survival time only about one year. Currently, docetaxel plus prednisone combo has become the first-line chemotherapy regimen for CRPC [3–5, 35–37]. Other therapeutic options include immunotherapy, targeted therapy, and chemotherapy [3–5, 35–37]. Yet the overall survival of CRPC patients has not been significantly improved over the years [3, 4]. Therefore, exploring of novel therapeutic targets and corresponding anti-CRPC agents is urgent needed [7].

Previous studies have supported HBO1 as a promising and important therapeutic oncotarget of human malignancies [14, 20–24, 38, 39]. MacPherson et al., have shown that HBO1 is vital for the maintenance of the leukemia stem cells [22]. Inactivation or silencing of HBO1 largely inhibited acute myeloid leukemia cell growth [22]. Gao et al., revealed that HBO1 expression is significantly elevated in osteosarcoma, and HBO1 depletion resulting in robust anti-osteosarcoma activity [25]. Zhong et al., have reported that overexpression of HBO1 is important for the progression of hepatocellular carcinoma [21]. Contrarily, CRISPR/Cas9-induced HBO1 knockout potently inhibited hepatocellular carcinoma cell growth in vitro and in vivo [21].

In the present study, we found that *HBO1* mRNA and protein expression is significantly elevated in human CRPC tissues and cells. Its expression is however relatively low in prostate epithelial tissues and cells. Moreover, shRNA-induced silencing of HBO1 resulted in robust anti-prostate cancer cell activity, inhibiting primary CRPC cell viability, proliferation, migration, and provoked apoptosis. On the contrast, ectopic overexpression of HBO1 further enhanced primary prostate cancer cell proliferation and migration. More importantly, HBO1 silencing, by intratumoral injection of HBO1 shRNA AAV, robustly suppressed pPC-1 xenografts in nude mice. Therefore, HBO1 is an important therapeutic oncotarget for CRPC.

WM-3835 is a potent, first-in-class and cell-permeable HBO1 inhibitor [20, 22, 25]. Studies have shown that WM-3835 reduced H3K14ac levels and inhibited growth and viability of AML cells [22]. WM-3835 remarkably inhibited osteosarcoma cell proliferation and migration, and provoking apoptosis [25]. Chen et al., reported that targeted inhibition of HBO1 by WM-3835 inhibited non-small cell lung cancer cell growth [20]. Furthermore, WM-3835 injection strongly suppressed different xenograft growth in mice [20–22, 25].

Here we provided evidence to support that WM-3835 is a promising therapeutic agent for CRPC. In the primary human prostate cancer cells that were derived from different CRPC patients, treatment with WM-3835 potently inhibited cell viability, colony formation, proliferation, cell cycle progression and in vitro cell migration. The HBO1 inhibitor provoked robust apoptosis activation in prostate cancer cells. It however failed to provoke significant cytotoxicity and apoptosis in human prostate epithelial cells. Daily *i.p*. injection of WM-3835 potently inhibited pPC-1 xenograft growth in nude mice, and no apparent toxicities were detected in WM-3835-treated mice. Thus, it will be of great translational value to explore the potential therapeutic effect of WM-3835 against CRPC or other advanced prostate cancers.

Docetaxel is still the first line chemotherapy for metastatic CRPC patients before or after abiraterone [40, 41]. However, its therapeutic efficacy is often limited due to the development of chemoresistance [40, 41]. One important finding of the present study is that treatment with WM-3835 sensitized docetaxel-induced cytotoxicity in primary CRPC cells. Thus, further studies will be needed to examine whether WM-3835 could sensitize CRPC cells to current therapeutic protocols, and to explore the possible underlying mechanisms.

Studies have suggested that targeting histone acetylation could be a valuable treatment option for CRPC. Joshi et al., have shown that CPT1A (the liver isoform of CPT1) promoted CRPC cell growth by supporting histone acetylation [42]. CPT1A knockdown inhibited histone acetylation [42]. Jin et al., reported that therapeutic inhibition of CBP/p300 blocked histone acetylation and inhibited CRPC cell growth [43]. Here we found that treatment with WM-3835 or HBO1 silencing inhibited H3-H4 acetylation (at multiple sites) and downregulated several pro-cancerous genes (*CCR2*, *MYLK*, *VEGFR2*, and *OCIAD2*) in primary CRPC cells. Moreover, H3-H4 histone acetylation and HBO1-dependent genes were downregulated in WM-3835-treated or HBO1-silenced xenograft tissues. Therefore, WM-3835-induced anti-CRPC activity could be due to inhibition on histone acetylation and silencing of cancer-promoting genes.

## Conclusion

Together, targeting HBO1 by WM-3835 robustly inhibited CRPC cell growth.

## Supplementary information

Author contribution form

aj-checklist FORM

Figure S1

## Data Availability

All data are available upon request.
