# Supplementary material for: A first-in-class HBO1 inhibitor WM-3835 inhibits castration-resistant prostate cancer cell growth in vitro and in vivo
Source: Cell Death Dis. 2023 Jan 28;14(1):67. doi: 10.1038/s41419-023-05606-5 (PMC9884225; doi:10.1038/s41419-023-05606-5)
Supplement: Supplementary file 1 — Author contribution form [file 41419_2023_5606_MOESM1_ESM.pdf]

DECLARATION OF CONTRIBUTIONS TO ARTICLE

ADMC

Manuscript Number: CDDIS-22-3291R

Journal Name: *Cell Death & Disease* (the 'Journal')

Proposed Title of the Contribution:

A first-in-class HBO1 inhibitor WM-3835 inhibits castration-resistant prostate cancer cell growth in vitro and in vivo (the 'Contribution)

Author(s):

Yuan-yuan Mi, Yu Ji, Lifeng Zhang, Chuan-yu Sun, Bing-bing Wei, Dong-jie Yang, Hong-yuan Wan, Xiao-wei Qi, Sheng Wu and Li-jie Zhu (the 'Authors')

For all *CDDis* articles, each person named as an author in the published version must be able to show he or she has contributed substantially to the article.

Authorship credit should be based on 1) substantial contributions to conception and design, acquisition of data, or analysis and interpretation of data; 2) drafting the article or revising it critically for important intellectual content; and 3) final approval of the version to be published. Authors should meet conditions 1, 2 and 3.

Any person who cannot be shown to have made a substantial contribution to the article cannot be listed as an author in the final version. The name of any person who is deemed to have made a minor contribution can, however, appear in the Acknowledgments section of the article.

Please complete the table below to indicate the contributions of all named authors to the manuscript.

Author Full Name:	Specification of Contribution to the Manuscript:
Yuan-yuan Mi	Functional assays in CRPC cells and normal epithelial cells, establishing mice xenografts, testing signalings and cell death/apoptosis, drafting the article and revising it, with final approval of the version submitted to the journal.
Yu Ji	Functional assays in CRPC cells and normal epithelial cells, establishing mice xenografts, testing signalings and cell death/apoptosis, drafting the article and revising it, with final approval of the version submitted to the journal.
Lifeng Zhang	Functional assays in CRPC cells and normal epithelial cells, establishing mice xenografts, testing signalings and cell death/apoptosis, drafting the article and revising it, with final approval of the version submitted to the journal.
Chuan-yu Sun	Functional assays in CRPC cells and normal epithelial cells, establishing mice xenografts, testing signalings and cell death/apoptosis, drafting the article and revising it, with final approval of the version submitted to the journal.
Bing-bing Wei	Functional assays in CRPC cells and normal epithelial cells, establishing mice xenografts, testing signalings and cell death/apoptosis, drafting the article and revising it, with final approval of the version submitted to the journal.
Dong-jie Yang	Functional assays in CRPC cells and normal epithelial cells, establishing mice xenografts, testing signalings and cell death/apoptosis, drafting the article and revising it, with final approval of the version submitted to the journal.
Hong-yuan Wan	Functional assays in CRPC cells and normal epithelial cells, establishing mice xenografts, testing signalings and cell death/apoptosis, drafting the article and revising it, with final approval of the version submitted to the journal.
Xiao-wei Qi	Study conception and design, and data analysis, Figure organization, involved in drafting the article and revising it critically for important intellectual content, and with final approval of the version submitted to the journal.
Sheng Wu	Study conception and design, and data analysis, Figure organization, involved in drafting the article and revising it critically for important intellectual content, and with final approval of the version submitted to the journal.
Li-jie Zhu	Study conception and design, and data analysis, Figure organization, involved in drafting the article and revising it critically for important intellectual content, and with final approval of the version submitted to the journal.

Please complete the table below to indicate the contributions of all named authors to the figures.

Figure 1:

Yuan-yuan Mi, Yu Ji, Lifeng Zhang, Chuan-yu Sun, Bing-bing Wei, Dong-jie Yang, Hong-yuan Wan, Xiao-wei Qi, Sheng Wu and Li-jie Zhu

Figure 2:

Yuan-yuan Mi, Yu Ji, Lifeng Zhang, Chuan-yu Sun, Bing-bing Wei, Dong-jie Yang, Hong-yuan Wan, Xiao-wei Qi, Sheng Wu and Li-jie Zhu

Figure 3:

Yuan-yuan Mi, Yu Ji, Lifeng Zhang, Chuan-yu Sun, Bing-bing Wei, Dong-jie Yang, Hong-yuan Wan, Xiao-wei Qi, Sheng Wu and Li-jie Zhu

Figure 4:

Yuan-yuan Mi, Yu Ji, Lifeng Zhang, Chuan-yu Sun, Bing-bing Wei, Dong-jie Yang, Hong-yuan Wan, Xiao-wei Qi, Sheng Wu and Li-jie Zhu

Figure 5:

Yuan-yuan Mi, Yu Ji, Lifeng Zhang, Chuan-yu Sun, Bing-bing Wei, Dong-jie Yang, Hong-yuan Wan, Xiao-wei Qi, Sheng Wu and Li-jie Zhu

Figure 6:

Yuan-yuan Mi, Yu Ji, Lifeng Zhang, Chuan-yu Sun, Bing-bing Wei, Dong-jie Yang, Hong-yuan Wan, Xiao-wei Qi, Sheng Wu and Li-jie Zhu

Figure 7 and 8

Yuan-yuan Mi, Yu Ji, Lifeng Zhang, Chuan-yu Sun, Bing-bing Wei, Dong-jie Yang, Hong-yuan Wan, Xiao-wei Qi, Sheng Wu and Li-jie Zhu

Signed for and on behalf of the Author(s):

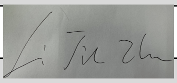

Print Name: Li-jie Zhu

Date: 2022-08-31
