# Supplementary material for: A first-in-class HBO1 inhibitor WM-3835 inhibits castration-resistant prostate cancer cell growth in vitro and in vivo
Source: Cell Death Dis. 2023 Jan 28;14(1):67. doi: 10.1038/s41419-023-05606-5 (PMC9884225; doi:10.1038/s41419-023-05606-5)
Supplement: Supplementary file 3 — Figure S1 [file 41419_2023_5606_MOESM3_ESM.pdf]

Figure S1: The uncropped blotting images of the study.

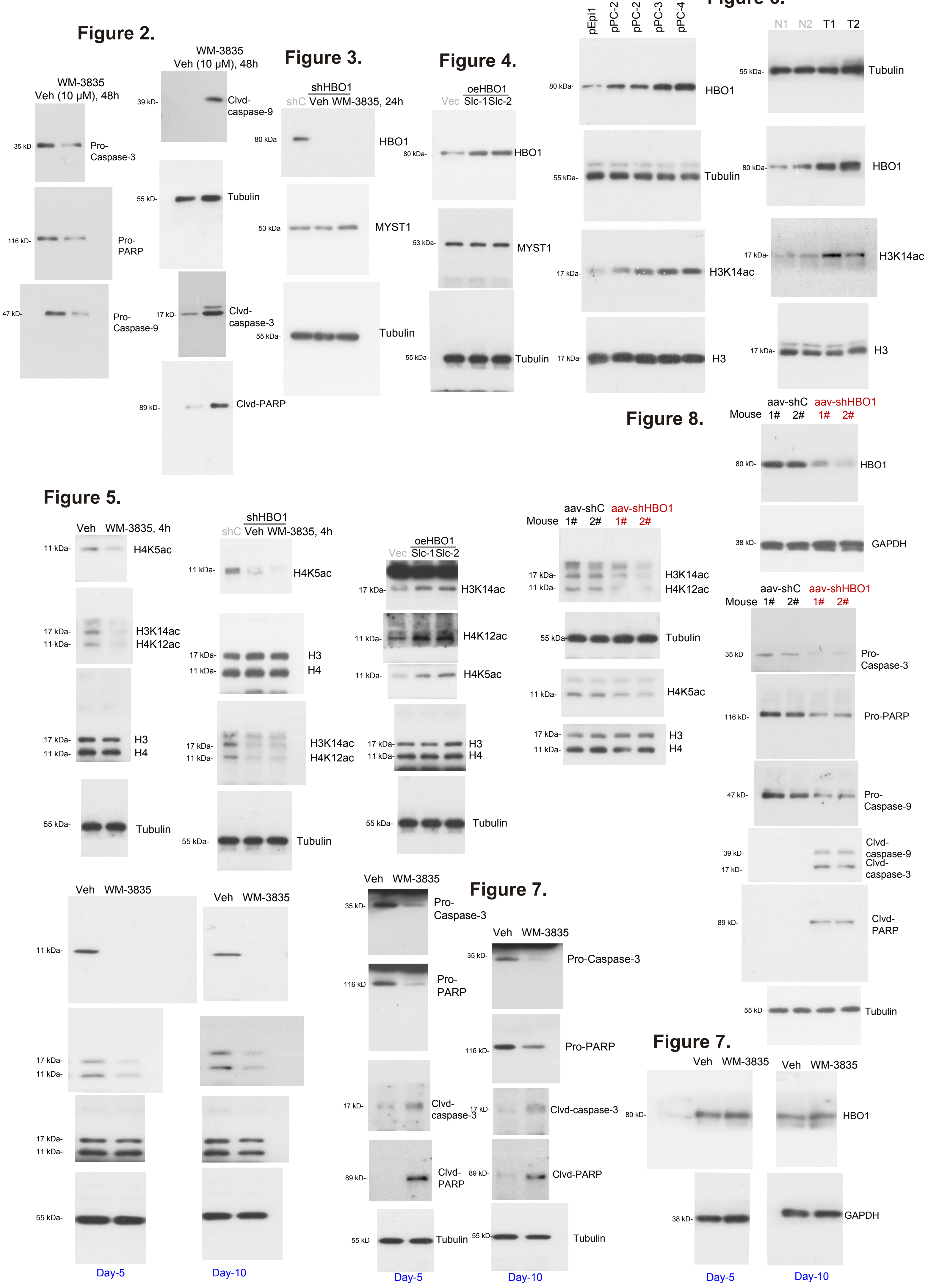

Figure S2.

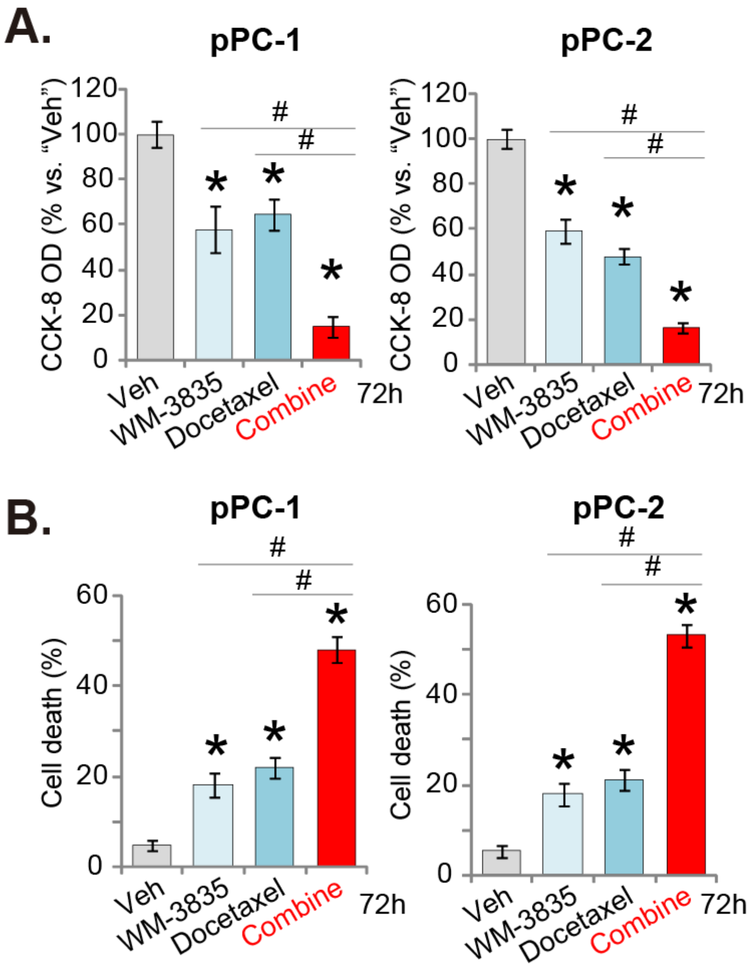

Figure S2. The primary human prostate cancer cells, "pPC-1/-2", were treated with vehicle control ("Veh"), WM-3835 (5 μ M), docetaxel (15 nM) or WM-3835 plus docetaxel combine ("Combine"). Cells were then cultured for additional 72h, cell viability (CCK-8 assay, **A**) and cell death (by testing medium LDH releasing, **B**) were measured. Data were expressed as the mean ± standard deviation (SD, n=5). ****P*** < 0.05 versus "Veh" group. $^{\#}$ ***P*** < 0.05. Data were expressed as the mean ± standard deviation (SD, n=5). In this figure, experiments were repeated five times, and similar results obtained.
